# Dr. Mary Putnam Jacobi

**Published:** 1906-07

**Authors:** 


### OBITUARY.

Dr. Mary Putnam Jacobi, of New York, wife of that distinguished physician, Dr. Abraham Jacobi, and herself one of the most distinguished women physicians in America, died at her home June 10, 1906, after a prolonged illness, aged 63 years. She was born in London, August 31, 1842, and was the daughter of George Palmer Putnam, founder of the great publishing house of that name. She first graduated as doctor of pharmacy in New York and then entered the Woman's Medical College of Philadelphia in 1861. After graduation she served a year as interne in a Boston hospital and after two years' private practice entered the School of Medicine at Paris in 1866, being the first woman ever admitted to that institution, and from which she graduated in 1871, receiving the second prize for her graduation thesis.

She soon returned to this country and took up once more the practice of her profession in New York. She was the first woman physician admitted to fellowship in the New York Academy of Medicine and was, likewise, the first woman sent as a delegate to the Medical Society of the State of New York. She became professor in the Woman's Medical College of the New York Infirmary, where she taught for ten years and afterward became a teacher at the New York Post-Graduate Medical School and Hospital. She was physician to the out-patient department of Mt. Sinai Hospital and attending physician to St. Mark's Hospital.

Dr. Mary Putnam, in 1873, married Dr. Abraham Jacobi of New York. In 1874 she was elected president of the Association for the Advancement of the Medical Education of Women, a society which she organised. She was gifted as a speaker and writer and devoted much time to the improvement of the education of her sex. Her medical writings alone comprise over forty papers, contributed to periodicals and encyclopedias. In 1876, she was awarded the Boylston prize at Harvard for an essay entitled "The Question of Rest for Women during Menstruation." This remarkable woman in many ways left her impress on the medicine of the period in which she lived.

